# Whole-Body Vibration Does Not Seem to Affect Postural Control in Healthy Active Older Women

**DOI:** 10.1155/2018/5798265

**Published:** 2018-04-23

**Authors:** P. S. C. Gomes, M. O. Campos, L. F. Oliveira, R. G. T. Mello, I. A. Fernandes

**Affiliations:** ^1^Laboratory Crossbridges, Physical Education and Sport Institute, Universidade do Estado do Rio de Janeiro, Rio de Janeiro, RJ, Brazil; ^2^School of Physical Education and Sports, Universidade Federal do Rio de Janeiro, Rio de Janeiro, RJ, Brazil; ^3^Naval Academy, Brazilian Navy, Rio de Janeiro, RJ, Brazil; ^4^Biomedical Engineering Program (COPPE), Universidade Federal do Rio de Janeiro, Rio de Janeiro, RJ, Brazil

## Abstract

**Objective:**

This study investigated the acute residual effects induced by different frequencies of whole-body vibration (WBV) on postural control of elderly women.

**Design:**

Thirty physically active elderly women (67 ± 5 years) were randomly divided into three groups: two experimental groups (high WBV frequency: 45 Hz and 4 mm amplitude, *n* = 10; low WBV frequency: 30 Hz and 4 mm amplitude, *n* = 10) and one control group (*n* = 10), with no treatment. The participants were first subjected to stabilometry tests and were then guided through three sets of isometric partial squats for 60 s while the WBV stimulation was applied. The control group was subjected to the same conditions but without the WBV stimulation. The participants were again subjected to body balance tests immediately following the end of the intervention period and again at 8, 16, and 24 min. To measure body sway control, three 60 s tests were performed at 10 s intervals for each of the following experimental conditions: (1) eyes opened and (2) eyes closed. The following variables were investigated: the average velocity of the displacement of the centre of pressure in the anterior-posterior and medial-lateral planes as well as in the elliptical area.

**Results:**

A 3 (condition) × 5 (test) two-way repeated-measures ANOVA did not identify significant differences in the stabilometric variables, regardless of group, time, or experimental condition.

**Conclusions:**

The effect of WBV, regardless of the stimulation frequency, did not have a significant effect immediately after or up to 24 minutes after vibration cessation, on the variables involved in the control of postural stability in physically active elderly women.

## 1. Introduction

Recognized as a public health problem among the elderly [1], falls are directly associated with physical impairments, psychological disabilities, and, consequently, losses of independence and quality of life [2, 3]. Of the key risk factors associated with falls among this age group, the decline in the balance control is notable [4, 5].

This condition is a consequence of aging because it is associated with deterioration in the neuromuscular and sensory systems as well as the centralized processing of sensory signals [6–8]. However, many studies have shown that exercise training can induce neuromuscular adaptations, optimize balance, and reduce the functional performance declines that are typical of aging [9–11]. Recent evidence suggests that medium- and long-term exercise combined with the application of whole-body vibration (WBV) are new options that can contribute to improved balance control [12–14].

Recently, young adult males and females submitted to WBV improved acutely static and dynamic balance [15].

To our knowledge, only one study investigated the residual acute effects of a single session of WBV in postural control in the elderly [16]. The increased proprioceptive feedback generated by a vibratory stimulation might temporarily increase neural stimulation to the muscles, thereby allowing for a greater recruitment and synchronization of the motor units and subsequent improvements in motor control [17, 18]. Prior to the suggestion of this mechanism, some authors suggested this type of intervention as a coadjuvant activity (whether preliminary or concomitant) for training that involves functional activities and tasks that require bodily stability control [17, 18].

Commonly, studies have used training routines, including the use of partial isometric squat concomitant to vibratory stimulus [13–16]; however, there is a large variability in the intensity of the stimuli used in the studies, including protocols that use 2 to 40 Hz [19, 20]. In a recent review [20], it was identified that most studies in the elderly population have contemplated the implementation of a program with a frequency of 30–40 Hz for the optimization of variables focused on muscle performance. The typical volume found, considering the duration of the stimulus VCT, included protocols from 30 to 60 s and 1 to 3 stimuli with 60 s rest [20]. For prescription purposes, however, evidence based information is not available concerning how to manipulate the variables that control stimulation intensity (i.e., vibration frequency and amplitude) to affect balance.

Therefore, this study investigated the acute residual effect induced by different WBV stimulation frequencies on postural control of elderly women and determined the duration of this effect.

## 2. Materials and Methods

### 2.1. Participants

The sample consisted of 30 women volunteers who were program participants at the Open University of the Third Age at Gama Filho University of Rio de Janeiro, Brazil. The study only included volunteers who were 60 years of age or older and who signed an informed consent form in accordance with the ethical standards established by the National Health Council (196/96). The study was approved by the ethics committee from Gama Filho University (protocol 0023.0.312.000-09).

The exclusion criteria were (1) the use of any auxiliary walking aid (e.g., crutches, canes, and tripod support); (2) infirmities or neurological sequelae such as a deteriorating cognitive state; (3) musculoskeletal problems (e.g., a history of back pain, acute pelvic inflammation, or fracture) that would prevent participants from performing the test protocols and study exercises; (4) any health condition that contraindicates vibration exercises (e.g., kidney or gall stones, peripheral vascular disease, metabolic diseases, the use of prosthetics, orthotics, or both, pacemakers, stents, or bypasses); (5) a history of problems related to the vestibular system; or (6) a history of falls and musculoskeletal injuries over the 6 months prior to the study.

The participants were randomly divided into three independent groups: (1) high-frequency vibration (VIB A; *n* = 10, age = 67 ± 3 years, height = 151.6 ± 3.7 cm, and body mass = 66.0 ± 6 3 kg); (2) low frequency vibration (VIB B; *n* = 10, age = 67 ± 6 years, height = 157.0 ± 6.7 cm, and body mass = 69.5 ± 11.0 kg); and (3) control (CON; *n* = 10, age = 68 ± 6 years, height = 156.7  ± 4.7 cm, and body mass = 65.5  ± 10.8 kg). All participants were classified as physically active based on the International Physical Activity Questionnaire (IPAQ, long form, usual week, Version 8) [21].

### 2.2. General Procedures

The study consisted of two visits between 24 h and 72 h apart. The purpose of the first visit was to familiarize participants with stabilometry and the experimental procedures. All procedures were conducted at the same time of the day to avoid possible circadian rhythm influences on the dependent variables.

### 2.3. Experiment and Control Conditions

All participants initially underwent the stabilometric tests and were then randomly divided into the VIB A, VIB B, or control groups. For condition VIB A, the vibration stimulation was performed on a commercial use platform (Physio-Plate Med, Globus Italia, Codognè, Italy) at a frequency of 45 Hz and an amplitude of 4 mm. For condition VIB B, the volunteers were subjected to an oscillation frequency of 30 Hz. In both VIB A and VIB B, participants were subjected to three sets of vibration stimulation lasting 60 s each. Intervals of 60 s were applied between the sets.

The participants were subjected to a set of vibrations in a partial isometric squat position with their knees flexed at 110° from the thigh to the leg. This angle was controlled using a goniometer positioned laterally at the dominant leg by the investigator. Participants' feet were positioned on the same line and separated by a distance approximately equivalent to the biacromial diameter. Participants were barefoot, and the platform completely supported the soles of their feet.

To determine the duration of the acute residual effect induced by the vibration, the participants were again subjected to the body sway control test immediately following the stimulation and then at 8, 16, and 24 min. CON group participants were subjected to the same procedures but without the vibration stimulation.

### 2.4. Body Sway Control

The participants completed the bipedal balance test with their eyes open (EO) or eyes closed (EC). Participants in the EO condition looked at a fixed point on the wall (a 5 cm diameter circle 2 meters away). Each participant stood on a portable force platform (AMTI-AccusWayPLUSS, Advanced Mechanical Technology, Inc., Watertown, USA) without shoes and abducted to 10° with their upper limbs relaxed along their bodies, with their heels 6 cm apart [22].

The stabilometric variables investigated included the average velocity of the displacement in the (1) medial-lateral direction (VMx), (2) anterior-posterior direction (VMy), and (3) elliptical area of displacement from the centre of pressure (EA). These tests were designed based on three 60 s trials with 10 s intervals in which the first minute was intended as an adaptation period for each situation analyzed (EO and EC). The order of the visual conditions (EO/EC) was randomized. The processing of stabilometric signals was performed using a routine developed in MATLAB (Version 7.0, Mathworks, Natick, USA). These procedures were adopted based on the findings of a preliminary study that analyzed the reliability of stabilometric measurements. The results of this preliminary investigation suggested that the stabilometric variables included in the present study would be reliable for the adopted procedures (intraclass correlation coefficient [ICC] = 0.76–0.98, coefficient of variation [CV] = 3.0%–21.2%; Technical Error of Measurements [TEM] = 4.9%–31.5%). Although based on the results of a preliminary study, the data analyses were performed based on the average between the second and third tests.

### 2.5. Data Analyses

The Shapiro-Wilk test was used to verify the normal data distribution, and Mauchly's test was used to verify sphericity.

A 3 (condition) × 5 (test) two-way repeated-measures ANOVA was used to analyze the differences between the experimental and control groups as well as between the averages of the balancing test and the initial measurements immediately after and 8, 16, and 24 minutes after the end of the intervention period for each stabilometric variable and visual conditions (EO and EC). For analyses in which the data sphericity assumption was violated, the significance levels were adjusted using the Greenhouse-Geisser epsilon.

When a significant *F* was observed, Bonferroni's post hoc test was used to determine pairwise differences. The results were considered significant at *p* ≤ 0.05.

A commercially available statistical package (SPSS for Windows, Version 17.0, SPSS Inc., USA) was used for all analyses.

## 3. Results and Discussion

Although a preliminary analysis using the Shapiro-Wilk test indicated that some variables were not normally distributed, an ANOVA was nevertheless selected because it is a sufficiently robust test [23].

**Figure 1:**
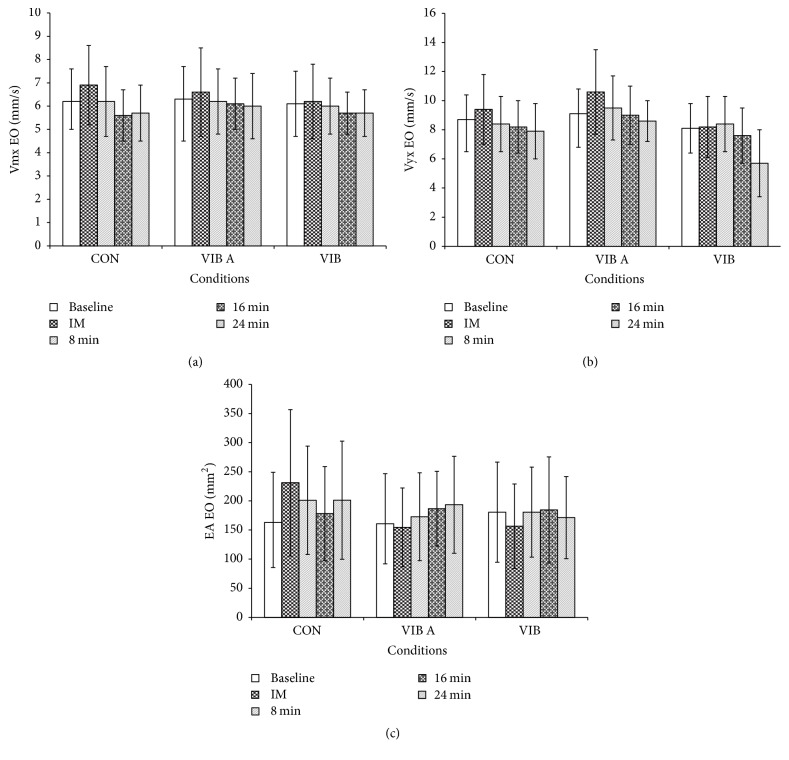
Mean values at baseline, immediately (IM) after the vibratory stimulus, and 8, 16, and 24 minutes after the vibratory stimulus. Mean velocity measures in the (a) medial-lateral direction (VMx; mm/s); (b) anterior-posterior direction (VMy; mm/s); and (c) elliptical area (EA; mm^2^), during eyes open (EO) condition.

**Figure 2:**
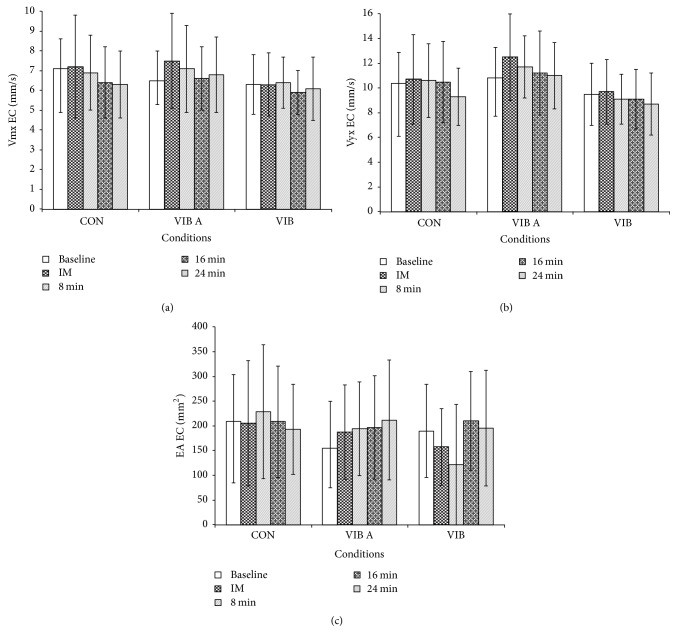
Mean values at baseline, immediately (IM) after the vibratory stimulus, and 8, 16, and 24 minutes after the vibratory stimulus. Mean velocity measures in the (a) medial-lateral direction (VMx; mm/s); (b) anterior-posterior direction (VMy; mm/s); and (c) elliptical area (EA; mm^2^), during eyes closed (EC) condition.

The 3 × 5 two-way repeated-measures ANOVA of the test factors did not identify any interactions between any of the variables investigated (VMx, VMy, and EA) for the measurements across both EO and EC conditions. Similarly, significant changes were not found between the initial measurements and those at different intervals following the intervention period for each experimental group (VIB A and VIB B) or the control group (Figures 1 and 2).

The results of the present study indicate that at both high and low frequencies, WBV did not significantly affect the variables involved on postural control of physically active elderly women as measured using stabilometry.

To our knowledge, only one study investigated the residual acute effects of a single session of WBV (35 Hz) in postural control in the elderly [16]. However, there were no observed effects of the vibrations on the balance control ability (before, immediately, and after 15 and 60 minutes) of the subjects, contributing to the findings of the present study. The experimental protocol used of the present study was similar to other studies using training routines, including the use of isometric partial squats while vibration stimulation was applied [13, 14, 16].

Consistent with the findings of the present study, other authors have reported the absence of effects on postural control using vibration stimulation [19, 24]. Schuhfried et al. [19] failed to identify any WBV effects (2.0–4.4 Hz, 3 mm) on the stability index, either immediately or 15 minutes following the vibration stimulation. However, the sample characteristics (individuals with multiple sclerosis) and frequencies of the vibration stimulation (2.0 to 4.4 Hz) strongly limited the generalizability of their findings.

Torvinen et al. [24] were also unable to observe changes to the body sway index among youths using a low intensity WBV stimulation (4 × 60 s, 15–30 Hz, 1.0 mm). In addition to the populations examined in these published studies, the presence of progressive fatigue in the soleus, gastrocnemius, and vastus lateralis muscles during the vibration stimulation deserves consideration; this fatigue was identified based on reductions in the frequency of electromyographic signals. In several studies indicating the influence of muscle fatigue on balance control [24–28], however, the authors [24] found no deleterious effects on postural control.

Contrary to these findings, other studies have shown favourable results with regard to the use of acute vibration to achieve improved balance control. However, the sample characteristics (youths and individuals with neuromuscular disorders) [17, 18, 25, 29], the vibration stimulation frequencies [17, 18], and the absence of information regarding the error associated with the measures used [17, 18, 25] severely limit comparisons and the generalizability of the findings.

The search for a dose-response relationship between the intensity of the vibration stimulation (i.e., vibration frequency and amplitude) and the induced effect on postural control has not been examined in other studies. However, other authors have corroborated the findings of this study and have reported the absence of a dose-response relationship among vibration stimulation intensity, the optimization of neuromuscular activation, and explosive strength [30–32]. Based on the previous literature and the results of the present study, no consensus exists regarding the occurrence of the residual acute effects induced by WBV on postural control. A portion of this inconsistency can be explained by the large variety of methodological procedures used in these studies, the diverse characteristics of the participants, and the lack of reporting with regard to measurement error. On the other hand, medium- and long-term WBV training is an intervention strategy that maximizes the variables involved in balance control and the other variables associated with the performance of functional tasks such as walking speed, mobility, and motor capacity [13, 14, 16]. Based on this evidence, this type of intervention has commonly been recommended for the elderly in an attempt to improve functionality and prevent falls [13, 14, 16].

## 4. Study Limitations

The results of the present study have to be interpreted with caution since our sample consisted of physically fit elderly women, somewhat different from most studies. Although the procedures used were very common in the literature, maybe a dynamic test would be more adequate to identify improvements with VCT. Unfortunately, a pilot study showed that tests using a dynamic platform were not reliable.

## 5. Conclusions

In conclusion, the acute effect of WBV, regardless of the frequency of the stimulation, did not significantly affect the variables involved in the control of postural stability (as measured using stabilometry) among asymptomatic and physically active elderly women. Given the findings of this study and the lack of consistency in the scientific literature, doubts remain regarding the occurrence of the acute residual effects induced by WBV on body balance. Therefore, randomized, controlled clinical trials should be conducted to better define the acute effects associated with the implementation of WBV on balance control and its associated mechanisms.

## Data Availability

The data used in the present investigation is kept in electronic files at the Laboratory Crossbridges, Physical Education and Sports Institute, Universidade do Estado do Rio de Janeiro, and available for download if necessary, upon request to the primary investigator.
